# A Novel 2.3 Mb Microduplication of 9q34.3 Inserted into 19q13.4 in a Patient with Learning Disabilities

**DOI:** 10.1155/2012/459602

**Published:** 2012-11-13

**Authors:** Shalinder Singh, Fern Ashton, Renate Marquis-Nicholson, Jennifer M. Love, Chuan-Ching Lan, Salim Aftimos, Alice M. George, Donald R. Love

**Affiliations:** ^1^Diagnostic Genetics, LabPlus, Auckland City Hospital, P.O. Box 110031, Auckland 1148, New Zealand; ^2^Genetic Health Service New Zealand-Northern Hub, Auckland City Hospital, Private Bag 92024, Auckland 1142, New Zealand; ^3^School of Biological Sciences, University of Auckland, Private Bag 92019, Auckland 1142, New Zealand

## Abstract

Insertional translocations in which a duplicated region of one chromosome is inserted into another chromosome are very rare. We report a 16.5-year-old girl with a terminal duplication at 9q34.3 of paternal origin inserted into 19q13.4. Chromosomal analysis revealed the karyotype 46,XX,der(19)ins(19;9)(q13.4;q34.3q34.3)pat. Cytogenetic microarray analysis (CMA) identified a *~*2.3Mb duplication of 9q34.3 → qter, which was confirmed by Fluorescence *in situ* hybridisation (FISH). The duplication at 9q34.3 is the smallest among the cases reported so far. The proband exhibits similar clinical features to those previously reported cases with larger duplication events.

## 1. Clinical Report

The proband was born prematurely at 35 weeks gestation with a birth weight of 2040 g. She required nasogastric tube feeding during the first week of life. During infancy, she was investigated for hypotonia and associated plagiocephaly; a brain MRI scan showed no abnormalities. She also had difficulties swallowing solids until the age of 2 years with ongoing tendency to drooling and keeping her mouth open. She walked at 2 years and 3 months of age. Her speech began developing at around that time. At school, she demonstrated age appropriate reading and writing skills, but required additional help in maths. However, the degree of her learning difficulty was minimal and psychometric assessment was not deemed to be necessary. She was also noted to have difficulties in gross motor and particularly fine motor skills and required assistance from an occupational therapist. An ophthalmic assessment at 16 years of age demonstrated myopia, with visual acuity of 6/24 in the right eye and 6/12 in the left eye. Fundoscopy revealed the presence of pigmentary changes in both posterior poles.

**Figure 1:**
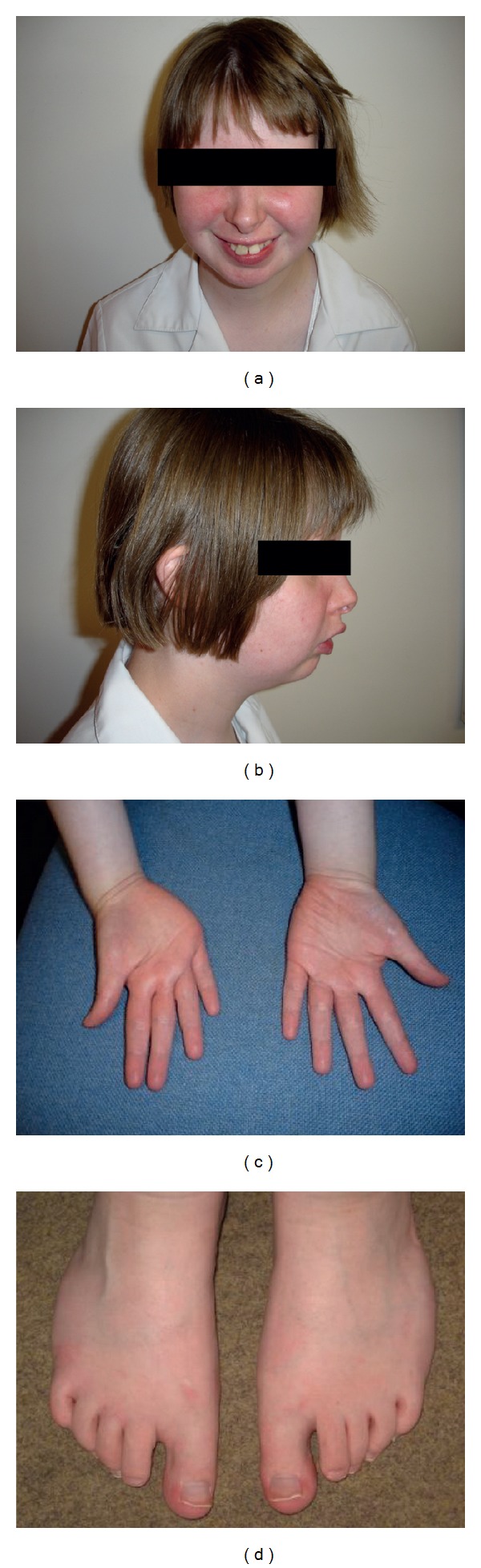
Clinical features of the patient at the age of 16.5 years. Frontal view (a) shows the short philtrum. Lateral view (b) shows mild micrognathia. (c) shows distal tapering of the fingers with radial clinodactyly of the middle three fingers, and (d) shows Long halluces, curly toes, and bilateral hallux valgus.

She was reviewed at the genetics clinic at 16.5 years of age. At that time, she was continuing to make good academic progress although she was receiving some input from the learning support unit attached to her school. Her height was at the 50th centile, weight at the 25th centile, and head circumference between the 25th and 50th centiles. Facial dolichocephaly and asymmetry were noted. The eyes were mildly deep set. She had a short philtrum and mild microganthia (Figures 1(a) and 1(b)), with a high arched palate. There was distal tapering of the fingers with radial clinodactyly of the middle three fingers (Figure 1(c)). She had long halluces, curly toes, and bilateral hallux valgus (Figure 1(d)). A mild scoliosis was also noted.

## 2. Chromosome Analysis

Conventional G-banded chromosome analysis was performed on peripheral blood samples taken from the proband and her parents. 

Genome-wide copy number analysis was determined from genomic DNA samples using the Affymetrix Cytogenetics Whole-Genome 2.7 M array, according to the manufacturer's instructions. Regions of copy number change were calculated using the Affymetrix Chromosome Analysis Suite software (ChAS) v.1.0.1 and interpreted with the aid of the UCSC genome browser (http://genome.ucsc.edu/; Human Mar. 2006 (hg18) assembly).

**Figure 2:**
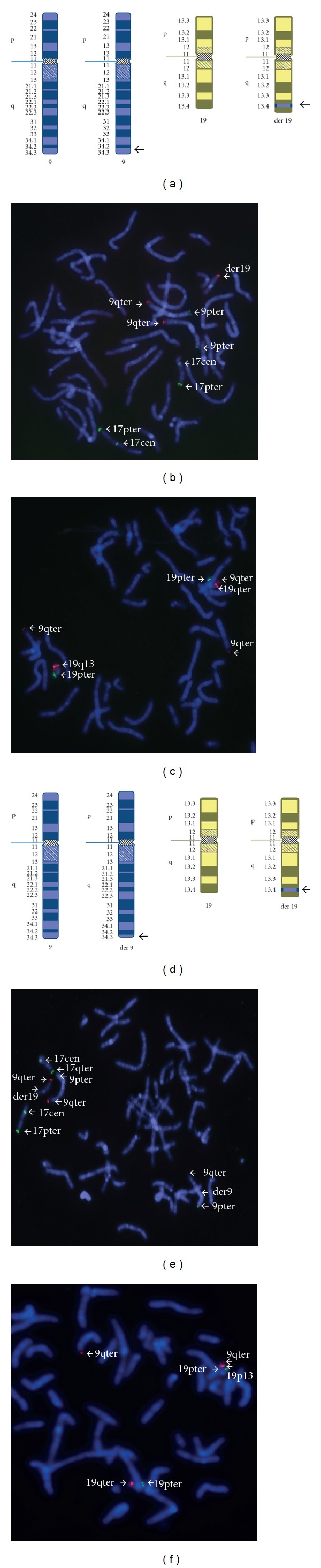
Cytogenetics and FISH analysis of proband and father. ((a)–(c)) and (d-f) show the analysis of the proband and father, respectively. Ideograms of chromosomes 9 and 19 show that part of region 9q34.3 is inserted into region 19q13.4 in the proband (a), and the father is a carrier of a balanced insertional translocation (panel d). FISH analysis used probes for 9pter (305J7-T7), 9qter (D9S325), 19pter (129F16/SP6), 19qter (D19S238E), 19q13 (GLTSCR1/GLTSCR2/CRX), while 17cen and 17q used control probes ((b)–(c) for the proband, and panels (e)–(f) for the father). These panels confirm that the part of region 9q34.3 is inserted into region 19q13.4. The subtelomeres of chromosome 19 were intact (the probes for 19pter (129F16/SP6) and 19qter (D19S238E) were used; image not shown).

**Figure 3:**
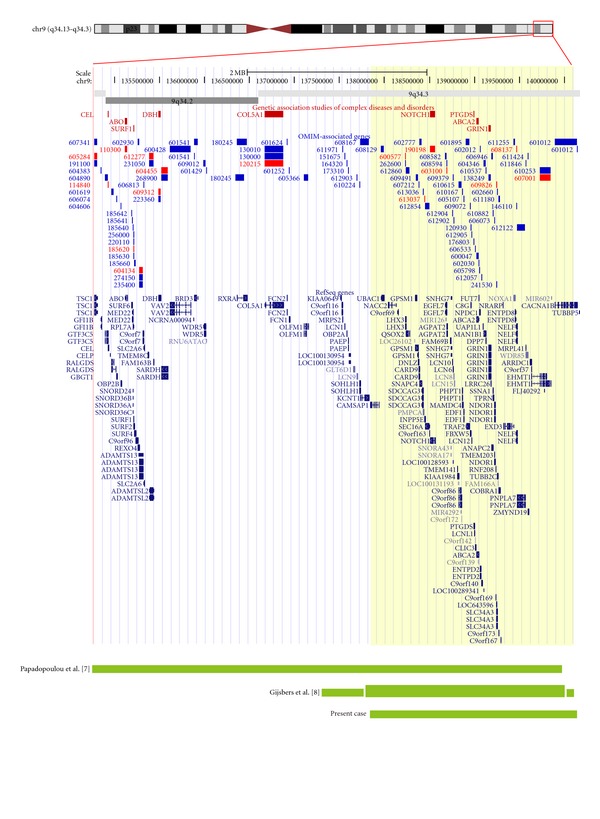
Location and extent of 9q34 duplications. UCSC Genome Browser (March 2006 (hg18) assembly) view of the chromosomal region 9q34.13-q34.3 (chr9:134,776,210-140,171,337) is shown, together with Refseq, OMIM, and GAD genes. The bottom panel shows the location and extent of the 9q34.3-qter region of the patient described here, and of other cases reported in the literature. Note: the region highlighted in yellow is the ~2.3 Mb region duplicated in our case, and the thicker green block represents the triplicated region reported by Gijsbers et al. [8].

Chromosomal analysis showed a female karyotype 46,XX,der(19)ins(19;9)(q13.4;q34.3q34.3) for the proband (Figure 2(a)). The father's karyotype was 46,XY,ins(19;9)(q13.4;q34.3q34.3) (Figure 2(d)) and the mother's karyotype was normal (data not shown). The array revealed a terminal duplication of approximately 2.3 Mb at 9q34.3, and the proband's molecular karyotype was arr 9q34.3(137,864,059-140,171,337)x3 (Figure 3; UCSC Genome Browser-NCBI Build 36, Mar. 2006 assembly).

FISH confirmed that a segment of region 9q34.3 was inserted into the region 19q13.4 using the locus-specific probe D9S325, with two signals on the chromosome 9 homologues present in the proband (Figures 2(b) and 2(c)). FISH using the probe specific for the 19q terminal region confirmed that the subtelomeres of the derivative chromosome 19 were intact. FISH findings from the father demonstrated an apparently balanced translocation: part of region 9q34.3 was inserted into 19q13.4, thus confirming the parental origin of the derivative chromosome 19 (Figures 2(e) and 2(f)). The duplicated region encompasses approximately 92 genes, which are likely to contribute to the proband's phenotypic features.

## 3. Discussion

Patients with 9q duplications have overlapping features, which include variable degrees of developmental delays, learning or intellectual deficits, facies characterised by dolicocephaly, asymmetry, deep set eyes or small palpebral fissures, high arched palate, micrognathia and digital anomalies including arachnodactyly, camptodactyly and clinodactyly. Furthermore, the finding of long halluces appears to be a common and distinctive feature in patients with a pure duplication, although many other reported cases carry copy number changes other than 9q duplications [1–8].

In this study, we report a small ~2.3 Mb duplication of 9q34.3 detected by CMA. Our patient displayed dolicocephaly and facial asymmetry, mildly deep-set eyes, short philtrum, mild microganthia, high arched palate, clinodactyly, mild scoliosis, mild myopia, and digital anomalies. A comparison of phenotypic anomalies of our patient with previously reported cases is summarised in Table 1. 

Recently, Gijsbers et al. [8] reported a 16-year-old girl with a triplication and duplications in the 9q34.3 region. The authors noted that the clinical features of their proband overlapped with those in one previous report [2], which was a “pure” 9q34.3 duplication case. The same dysmorphic features are shared with the proband reported here, but feeding difficulties, scoliosis, and severe mental retardation are absent. The more severe phenotype reported by Gijsbers et al. [8] may be attributed to a larger ~2.9Mb region of duplicated and triplicated subregions (chr9q34:137,265,834-140,207,437) that encompasses approximately 100 genes. In our case, approximately 92 genes are duplicated in a ~2.3 Mb region (chr9q34.3 : 137,864,059-140,171,337).

Of the genes contained within the duplicated region detected in our patient, eleven are present in the Online Mendelian Inheritance in Man (OMIM; http://www.ncbi.nlm.nih.gov/omim) morbid map, and of these, all but *NOTCH1* are associated with autosomal recessive disease and homozygosity for terminating mutations (Table 2). As a consequence, these OMIM genes do not appear to play a role in the clinical phenotype reported here which is likely to be caused by gene overexpression, due to the increased copy number of the 2.3 Mb region of chromosome 9, rather than haploinsufficiency.

In the mouse, upregulation of *NOTCH* activity appears to be associated with an increase in the number of interneuronal contacts and the cessation of neurite growth [9]. In addition, the *NOTCH* signalling pathway plays a pivotal role in embryo development. It is likely, given the mathematical modelling undertaken by Raya et al. [10], that increased expression of *NOTCH1* would have an impact on the level of NOTCH1-associated subcomplexes, and hence alter developmental and physiological outcomes. That *NOTCH1* over-expression may be the principal underlying gene responsible for the phenotype of our patient remains speculative at this stage. It is also possible that the site of insertion on chromosome 19 may affect the expression of chromosome 19 genes, which may play a role in the phenotype reported here. Unfortunately, the array data does not provide any clues regarding the specific site of insertion on chromosome 19.

In summary, the proband reported here is a new addition to the rare collection of dup 9q34 cases. Our patient has developed a mild form of the clinical features described in other 9q34 cases, possibly due to the smaller affected region. Patients with shorter dup 9q34 tend to have a better prognosis and would benefit from special education with input from their parents [2]. It is hoped that with increased reporting of similar cases, dosage changes and breakpoints in this region can be more clearly correlated to phenotypic features to aid genetic counselling and medical management. 

## Figures and Tables

**Table 1:** Clinical features in patients with duplication and/or triplication of the 9q34 region.

Cytogenetics	Hou and Wang [1] dup 9q32→q34.3	Allderdice et al. [2] dup 9q34.1→q34.3	Spinner et al. [3] and Youngs et al. [4]^a^ dup 9q33.3→qter	Mattina et al. [5] dup 9q34.1→qter	Gawlik-Kuklinska et al. [6] dup 9q34.1→q34.3	Papadopoulou et al. [7] dup9q34.1→q34.3	Gijsbers et al. [8] dup and trip 9q34.3	Present case dup 9q34.3→qter
Size of 9q duplication determined by molecular karyotype	Undetermined	Undetermined	13.79 Mb	Undetermined	7.26 Mb	~5–5.8 Mb	~0.53 Mb duplication; ~2.4 Mb triplication	~2.3 Mb
Other chromosomal anomalies	Null	Null	Del 12p13.33	Dup 21pter→q22.1	Null	Dels 15q21.2-15q21.3; 15q22.31-15q23; 15q25.1-15q25.2	Null	Null

Clinical features								

Dolicocephaly	+	+	+	∗	+	−	∗	+
Facial asymmetry	∗	+	+	∗	+	−	+	+
Deep-set eyes/small palpebral fissures	+	+	+	+	+	−	+	+
Beaked nose	+	+	+	+	−	+	−	−
High arched palate	+	+	+	+	∗	+	−	+
Micrognathia/ retrognathia	+	+	+	∗	+	+	+	+
Arachnodactyly/ camptodactyly	+	+	+	+	+	+	+	−
Long halluces	∗	+	+	+	+	+	∗	+
Scoliosis	∗	+	+	∗	+	−	−	+
Low birth weight	+	+	+	−	−	+	−	+
Hypotonia	+	+	+	+	+	+	−	+
Failure to thrive	+	+	+	∗	+	−	−	−
Cardiac defects	+	+	+	+	−	+	−	−
Developmental delay/intellectual disability	+	+	+	+	+	+	+	+

^a^Youngs et al. [4] is an 18-year follow-up report on an infant with dup 9q34 originally reported by Spinner et al. [3].

*Not reported or observed from published photographs.

**Table 2:** Duplicated region and OMIM genes.

OMIM	Protein	Gene	Disorder	Molecular genetics
600577	LIM/homeodomain protein LHX3	LHX3	Combined pituitary hormone deficiency-3	Homozygosity for intragenic deletion/nonsense mutation
613037	Inositol polyphosphate-5-phosphatase	INPP5E	Joubert syndrome 1	Homozygosity for mutations in the INPP5E gene that lead to decreased phosphatase activity
			Mental retardation, truncal obesity, retinal dystrophy, and micropenis	Homozygous nonsense mutation detected in the INPP5E gene
607212	Caspase recruitment domain-containing protein 9	CARD9	Autosomal recessive form of familial chronic mucocutaneous candidiasis	Homozygous nonsense mutation in the CARD9 gene
190198	Notch, Drosophila, homolog of, 1, translocation associated Notch homolog; *NOTCH1 *	*NOTCH1*	Aortic valve disease	Heterozygosity for nonsense/frameshift mutations
			Leukemia, T-cell acute lymphoblastic	
603100	1-Acylglycerol-3-phosphate O-acyltransferase 2	AGPAT2	Lipodystrophy, congenital generalised, type 1; CGL1	Homozygous or compound heterozygous mutations
613354	Taperin	TPRN	Autosomal recessive nonsyndromic deafness-79	Homozygous truncating mutations
604346	Mannosidase, alpha, class 1B member 1	MAN1B1	Mental retardation, autosomal recessive 15	Homozygous mutations
138249	Glutamate receptor, ionotropic, N-methyl D-aspartate 1	GRIN1	Mental retardation, autosomal dominant 8	Missense mutation; in-frame duplication of codon 560
609826	Solute carrier family 34 (sodium/phosphate cotransporter), member 3	SLC34A3	Hypophosphatemic rickets with hypercalciuria	Homozygous single-nucleotide deletion
608137	Nasal embryonic luteinizing hormone-releasing hormone factor	NELF	Hypogonadotropic hypogonadism	A thr480-to-ala mutation in the NELF gene
607001	Euchromatic histone methyltransferase 1	EHMT1	Kleefstra syndrome	Heterozygous nonsense/frameshift mutation, in the EHMT1 gene; terminal deletions, interstitial deletions, derivative chromosomes, and complex rearrangements

The entries in this table were taken from the OMIM database (http://www.ncbi.nlm.nih.gov/omim).
